# Acceptability of a Randomized Trial of Anti-depressant Medication or Interpersonal Therapy for Treatment of Perinatal Depression in Women with HIV

**DOI:** 10.1007/s10461-023-04264-0

**Published:** 2024-02-14

**Authors:** M. Bridget Spelke, Eunice Okumu, Nzi R. Perry, Bryan S. Blette, Ravi Paul, Crystal E. Schiller, J. M. Ncheka, Margaret P. Kasaro, Joan T. Price, Samantha Meltzer-Brody, Jeffrey S. A. Stringer, Elizabeth M. Stringer

**Affiliations:** 1 Department of Obstetrics and Gynecology, University of North Carolina School of Medicine, Chapel Hill, USA; 2 University of North Carolina – Global Projects Zambia, 348 Independence Ave, Lusaka, Zambia 10101; 3 Social and Behavioral Science Core, Center for AIDS Research, University of North Carolina, Chapel Hill, USA; 4 Department of Biostatistics, Vanderbilt University Medical Center, Nashville, USA; 5 Department of Psychiatry, University of Zambia School of Medicine, Lusaka, Zambia; 6 Department of Psychiatry, University of North Carolina School of Medicine, Chapel Hill, USA

**Keywords:** Interpersonal therapy, Depression, Zambia, Antidepressant medication, Low resource setting, HIV

## Abstract

Postpartum depression (PPD) affects nearly 20% of postpartum women in Sub-Saharan Africa (SSA), where HIV prevalence is high. Depression is associated with worse HIV outcomes in non-pregnant adults and mental health disorders may worsen HIV outcomes for postpartum women and their infants. PPD is effectively treated with psychosocial or pharmacologic interventions; however, few studies have evaluated the acceptability of treatment modalities in SSA. We analyzed interviews with 23 postpartum women with HIV to assess the acceptability of two depression treatments provided in the context of a randomized trial. Most participants expressed acceptability of treatment randomization and study visit procedures. Participants shared perceptions of high treatment efficacy of their assigned intervention. They reported ongoing HIV and mental health stigma in their communities and emphasized the importance of social support from clinic staff. Our findings suggest a full-scale trial of PPD treatment will be acceptable among women with HIV in Zambia.

**Supplementary Information:**

The online version contains supplementary material available at 10.1007/s10461-023-04264-0.

## Introduction

Despite being the primary contributor to global disability, depression (and mental health in general) is not yet a policy or funding priority in the Global South [1]. Postpartum mood disorders, beginning during pregnancy or the first month after delivery, have been under-recognized and under-treated but are increasingly recognized as a major health issue [2–4]. Postpartum depression (PPD) is thought to affect as many as one in five postpartum women in sub-Saharan Africa (SSA) and can have devastating consequences for both mother and child, especially when co-occurring with maternal HIV [5, 6]. Depression can severely reduce a woman’s ability to keep medical appointments or adhere to prescribed medications [7, 8]. It also reduces the length and quality of breastfeeding and can interfere with maternal-infant bonding [9]. While there is no worldwide shortage of new public health interventions being tested for mothers and children, mental health interventions have been left frustratingly behind [1].

Depression and anxiety have been treated with various forms of psychotherapy, medication, or a combination of both treatment modalities. In the few clinical trials that exist, neither treatment seems to show clear benefit over the others [10–12]. There are potential benefits and limitations for each type of treatment, particularly in places such as SSA where resources are limited and HIV seroprevalence is high. Among women with HIV on antiretroviral therapy (ART), adding an antidepressant medication may reduce medication adherence because of increased pill burden [13]. Behavioral and psychosocial interventions, such as cognitive behavioral therapy with integrated adherence counseling, can mitigate this risk and have demonstrated increased medication adherence in randomized trials among adults with both HIV infection and depression [14, 15]. A potential barrier to psychosocial interventions is the availability of mental health providers in countries with medical staff shortages [16]. However, task-shifting models of depression treatment show great promise in equipping primary care and mid-level providers with the training and supervision to safely and effectively manage depression in these settings [17, 18].

The acceptability of medical interventions is a substantial driver of adherence. Prospective studies that include antidepressant medication for the treatment of depression and anxiety are limited in Zambia. To better understand patients’ beliefs and attitudes towards depression and mental health treatments in the context of a clinical trial, we conducted in-depth, semi-structured interviews with participants enrolled in a pilot feasibility trial of antidepressant medication (ADM) versus interpersonal therapy (IPT) for treatment of depression or anxiety in HIV-infected postpartum women living in Lusaka, Zambia. The parent trial found that a randomized trial of these two treatments for depression was feasible in this setting and that both treatment modalities effectively treated depression at 6 months postpartum [19]. Here we present qualitative findings from a postpartum depression treatment study conducted to evaluate the acceptability of two common modalities for treating postpartum depression to improve adherence to ART; identify barriers and facilitators to trial participation; and the effects of mental health stigma in Zambia.

## Methods

The study protocol and interview guides were reviewed and approved by the University of North Carolina Institutional Review Board, the University of Zambia Biomedical Research Ethics Committee, the Zambia Medicines Regulatory Authority, and the Zambia National Health Research Authority before study initiation. Written informed consent was obtained from all participants in their preferred language (English, Bemba, or Nyanja).

### Study Design and Population

The perinatal depression study was conducted at Kamwala Health Clinic in Lusaka, Zambia between October 29, 2019, and September 8, 2020. Full trial procedures have been described previously and are summarized below [19]. Eighty women with HIV who met diagnostic criteria for either depression or anxiety were randomized postpartum to either ADM (sertraline) or IPT using a structured counseling manual that reviewed the following topics: (1) depression, (2) motherhood, (3) HIV treatment, (4) stigma, (5) self-stigma, (6) problem solving, (7) poverty, (8) relationships, (9) feeling alone, (10) grief and loss, and 11) disclosure. IPT was delivered by trained research nurses in individual 60-minute sessions at the study site. Nurses providing IPT completed an intensive 1-week training led by a licensed clinical psychologist and received one hour of virtual supervision weekly during the study.

Eligible participants were 18 years of age or older, had documentation of confirmed HIV-1 infection, were between 6 and 8 weeks postpartum, currently taking ART treatment, and had a diagnosis of anxiety or depression confirmed by Mini-International Neuropsychiatric Interview (MINI). Individuals who had taken an ADM in the 12 months before enrollment, were actively suicidal, had a known or suspected allergy or contraindication to sertraline, had taken other medications, or who had experienced a stillbirth or neonatal death in the pregnancy preceding enrollment were excluded. After undergoing informed consent and randomization, women were followed for 24 weeks with 12 scheduled visits. Participants were assessed for response to treatment at each visit with the Edinburgh Postpartum Depression Scale (EPDS), a 30-point scale of 10 questions, and the Clinical Global Impression—Severity (CGI-S), a subjective clinical assessment of disease severity [20, 21]. At the final study visit, women from both study arms were invited to participate in semi-structured interviews (SSIs).

### Data Collection

Women were invited to participate during their final study visit and provided separate written informed consent for the interviews. Recruitment stopped when thematic saturation was reached. Two female research assistants (RAs) trained in qualitative data collection independently conducted in-depth interviews in a private room reserved for research. Interview guides posed open-ended questions in the following topical areas: (1) health beliefs and attitudes towards depression and treatment for depression, (2) perceptions of mental health stigma in participants’ communities, (3) perceptions of participating in a research study for depression, and (4) attitudes towards randomized study intervention (Online Resources 1a & 1b). In addition, IPT arm participants were asked their thoughts on type of counseling sessions (i.e., individual or group counseling), mode of counseling sessions (in-person or by telephone), and frequency of the counseling sessions. ADM participants were asked about medication side effects, dose titration, and the frequency and necessity of clinic visits for medication management. Interviews lasted approximately 30–60 min and were audio-recorded with participants’ consent. Audio files were transcribed verbatim by an RA, translated to English, and analyzed by the University of North Carolina Center for AIDS Research Social and Behavioral Science (UNC CFAR SBS) Core team.

### Data Analysis

A thematic analysis approach was used to analyze the findings of this study. First, data was structurally coded using Dedoose, a web-based qualitative data management application. A preliminary structural codebook was developed and revised in collaboration with the UNC CFAR SBS Core analysts and the study research team members before uploading to Dedoose (Online Resource 2). Subsequently, two analysts independently coded transcripts using an iterative process to ensure coding consistency and agreement. Discrepancies in interpreting the data or applying codes were addressed and resolved throughout the coding process to ensure complete agreement among the analysts. The two analysts performed an inter-rater reliability test to confirm complete agreement. To support the analysis of relationships between codes, the analysts used memos and reviewed code co-occurrences within Dedoose. We summarize the findings according to predeveloped themes and the sub-themes generated from coding reports.

## Results

Thirty-one women completed the SSIs. Twenty-three of the 31 women (74%) had “sufficient” or “somewhat” sufficient responses that could be included in the analysis. Participants were considered to have “insufficient” responses if they mostly used closed-ended responses (i.e., “Yes”/“No”) on the SSIs. Of the 23 participants with interview data included in the analysis, 12 (52%) had been randomized to the ADM arm and 11 (48%) had been randomized to the IPT arm (Table 1).

**Table 1:** Baseline characteristics of participants interviewed and participants randomized to treatment, October 29, 2019–September 20, 2020 [19]

Characteristic	Overall	Interviewed and Included in analysis
	(*N* = 80)	(*N* = 23)
	n (%) or mean (SD)	n (%) or mean (SD)
Maternal age, years	29.7 (5.4)	30.7 (4.6)
Maternal education		
< 7 years	26 (33%)	9 (39%)
7–11 years	48 (60%)	11 (48%)
≥ 12 years	6 (8%)	3 (13%)
Married or living with partner	66 (83%)	21 (91%)
In poverty	52 (65%)	17 (74%)
Parity		
1	8 (10%)	1 (4%)
2–3	41 (51%)	10 (43%)
> 3	31 (39%)	12 (52%)
Comorbidities outside pregnancy^a^		
Any prior	5 (6%)	1 (4%)
Depression	1 (1%)	0
Antecedent delivery		
Infant birthweight	2900 (490)	3100 (400)
< 2500 g	15 (19%)	2 (9%)
Vaginal delivery	77 (96%)	23 (100%)
Breastfeeding		
Exclusive	74 (93%)	22 (96%)
Mixed	6 (7%)	1 (4%)
Years since HIV diagnosis	4.7 (3.9)	5.0 (3.9)
≤ 1 year	22 (28%)	5 (22%)
2–5 years	34 (44%)	12 (52%)
> 5 years	22 (28%)	6 (26%)
Missing	2	0
Years since ART initiation	4.1 (3.2)	4.9 (3.7)
≤ 1 year	23 (29%)	5 (22%)
2–5 years	36 (46%)	12 (52%)
> 5 years	19 (24%)	6 (26%)
Missing	2	0
Viral load		
< 40 copies/mL	59 (74%)	18 (78%)
40–1000 copies/mL	8 (10%)	1 (4%)
> 1000 copies/mL	13 (16%)	4 (17%)
EPDS at enrollment	13.9 (4.1)	13.9 (4.6)
≤ 13	41 (51%)	11 (48%)
> 13	39 (49%)	12 (52%)
MINI diagnosis at enrollment		
Anxiety	79 (99%)	23 (100%)
Depression	55 (69%)	13 (57%)
PTSD	25 (31%)	5 (22%)
Anxiety and depression	54 (68%)	13 (57%)
Experienced gender-based violence	30 (37%)	7 (30%)
Number of current life stressors	3.2 (2.1)	3.5 (2.3)
*IPT* interpersonal psychotherapy, *ADM* antidepressant medication, *ART* antiretroviral therapy, *EPDS* Edinburgh Postnatal Depression Scale, *MINI* Mini International Neuropsychiatric Interview, *PTSD* Post-traumatic stress disorder		

^a^ Comorbidities include patient report of chronic hypertension, diabetes mellitus, thyroid disease, and anemia

### Attitudes Toward Study Interventions and Procedures

#### Acceptability of Assigned Intervention

Participants from both arms were asked how they felt when they learned the arm to which they had been randomized. All ADM arm and almost all IPT arm participants responded to this question. Most participants randomized to ADM (10/12; 83%) and IPT (8/10; 80%) reported that the intervention was acceptable by expressing preference or impartiality towards their assigned intervention.

“Because I didn’t know anything, I just knew that this was a study so when they told me I would receive medication, I accepted. Everything is moving at the same pace, the medication and learning.” (ADM)

“…the one that was chosen for me is the one I was happy with.” (IPT)

A few patients expressed initial preferences toward one of the arms. One ADM arm participant reported a preference for counseling at first but later found the ADM arm acceptable. Two of the IPT arm participants reported strong feelings of acceptability towards the counseling arm, stating that it is the arm they “really wanted”.

“Well at first, I feel…mmm. What is it now? To take the what instead of counseling? But after now I get used to the ADM. I used to feel good so it’s better be in the ADM than have the counseling.” (ADM)

“I think the counseling one was better. I feel the medication one is not good…looking at the situation I was in, it’s better when you talk to someone.” (IPT)

#### Participant Perceptions of the Value and Efficacy of Assigned Treatments

Participants from both randomization arms were asked to describe how they were feeling since enrolling in the study, whether they perceived that the intervention was working, and whether anything happened that might have affected their mood. All participants expressed opinions of high efficacy towards the interventions while 7/11 (64%) of participants in the IPT arm specifically reported feeling better since joining the study. Participants in the ADM arm were asked how the medication made them feel, whether it had affected their ability to perform their daily activities and responsibilities, and whether it changed their normal behaviors. Participants from both arms who responded to these questions only cited positive perceptions of the assigned intervention.

ADM arm participants cited feeling “fine”, and that their ability to perform their daily duties and normal behaviors “remained the same.” Slightly less than half (5/12; 42%) of them reported that they didn’t experience anything unusual when taking their medication.

“I’ve been feeling very fine…The medication? Yeah, it has worked.” (ADM)

“I am feeling just fine. All the pressure I had, I now understand because pressure come and go in one’s life…Okay from the time I started counseling, I noticed that things have changed. My behavior, because I used to act like if something comes, before I even understand it, I would react sometime back. So, from the time I started counseling, I knew that a person should act like this such that if a situation comes, you need to understand it well and know which action to take. So, I see as though it has really helped me in this situation because it is different from the way things were before.” (IPT)

“Yeah at first, okay, I thought how am I going to cope with that? Then I find after I’ve taken it [medication], uh, I find no, no problem with me so I get used to it… Hm the [normal] behavior. Ah, they never changed. Just remained the same.” (ADM)

Most (10/11; 91%) IPT arm participants reported that they performed their regular duties and attended counseling sessions without any problems. One participant reported that she was able to share medication adherence lessons learned with her partner, which has made him adhere more to his antiretroviral medications than before.

“We learnt about poverty and we also learnt about HIV/AIDS… no, I managed to perform my normal duties, I managed to do everything… it didn’t affect my relationship with my partner, I was able to explain to him about what we were learning here and everything. He liked to skip days when taking his ARVs but now he takes them consistently because I always encourage him to do so.” (IPT).

“What I learnt in this study with the counselors that I find when I come here, especially as a married woman, I have learnt a lot. […] That feeling of keeping a grudge in your heart, especially knowing that your status is not okay. So, you need to know how to control yourself because the way I am right now, I have a baby, that baby to grow well and not get infected by the disease, I need to know how to keep myself, especially my baby. I even started teaching my friends… because when starting this program, when I started coming here, I sat down with them [family members] and he allowed me to start coming here so it didn’t affect me badly, no. It taught me a lot, whatever I learnt, when I experienced a bad issue, I would know how to control myself so there wasn’t any problem.” (IPT)

Two IPT arm participants expressed feelings of gratitude towards the program, with one of them further citing their partner’s support and gratitude for the sake of their newborn baby:

“From the time I joined, when we started, I feel fine and again I am so happy because I have learnt a lot, my heart is at peace as compared to the way I was before I joined the study, so I am grateful. […] I also informed him [partner]… I decided [to say] because the things that happen are plenty. So, I sat down and told my husband that ‘I go to Kamwala clinic where they offer counseling. The way I am, am positive. So, during counseling you find that someone has excessive thoughts. Sometimes we misunderstand each other, and I start to over think even now when I have kids. So, I agreed to join counseling so that they can teach me the way I can avoid certain things and move forward’…He was grateful, and he said it’s a good thing because you find that you are not well and you start thinking too much then to make matters worse if you are breastfeeding, you can affect the baby. So, he was grateful…they felt very good.” (IPT)

The other participant discussed challenges with depression such as isolation, aggressive behaviors, hopelessness, low self-esteem, changes in sleep pattern, and thoughts of death. She credited the program for providing the education, awareness, and counseling that helped her cope with depression. She reported challenging cognitive distortions, altering her behavioral patterns, regulating her emotions, and developing coping strategies. She also reported that the program inspired her to improve her quality of life and pursue a career in counseling. These behavior modifications reflect concepts of Cognitive Behavior Therapy (CBT) treatment for depression.

“Umm right now I’m very grateful for this program because it has really helped me a lot and it has also helped the people who were around me and especially my children […] since this program I became more aware of the people that I was hurting and the blame that I was putting on my life for no very good reason. […]You will think this one is living ok, but people are so depressed. Especially us women, African women. I don’t know what to say. But I’m very much grateful, I think I’ll be grateful for the rest of my life for this program. Because if it wasn’t for this program… now I’m able to be grateful for little, little things that I never could imagine. You know I’m grateful for the family that I have. But because of this program there are so many numerous things that I can mention that this program has helped me with. But this is the few that I can talk about.” (IPT)

#### Lessons Learned from Study Participation

Participants in the IPT arm were asked to describe what they learned in the counseling sessions, how the counseling affected their ability to perform their normal behaviors, and whether going for the counseling sessions affected their relationship with their partner, family members, or daily responsibilities. When asked if they learned anything from being in the study, approximately three-quarters of participants in both arms reported learning about depression, self-control, self-care, management of intrusive thoughts, the importance of reaching out for health care and medication adherence, as well as stigma awareness and resolution. Nearly all (10/11; 91%) IPT arm participants reported learning more specifically about HIV/AIDS and dealing with HIV diagnosis. Other lessons included caregiving and dealing with loss and grief.

More than half of participants in the ADM arm and half in the IPT arm reported identifying ways to control their emotions, manage intrusive thoughts, and care for themselves while struggling with depression.

“I learnt about not having excess thoughts, if anything happens, you need to relax and avoid over thinking. Even when am angry, I control myself because if you think too much, you can have problems like depression, so it encourages me…I used to think of anything but now because of this study, I think of relaxing. I found something good in this program, so I take it easy.” (ADM)

“I learnt how to take care of myself when I first started, my heart was heavy but now am free. Looking at the situation I was in, so I now know which path to take whenever I start to feel low.” (IPT)

#### Attitude Toward Clinic Visits

ADM arm participants were asked how they felt about coming to the clinic to pick up their medicine and hypothetical changes in the frequency of clinic visits. IPT arm participants were asked how they felt about coming to the clinic for counseling sessions, their thoughts on the number of counseling sessions, and issues they had getting to the clinic for the counseling sessions.

All participants in the ADM arm expressed positive attitudes towards medications provided through clinic visits, mostly stating that they “felt/feel just fine” about coming to the clinic to pick up their medication and the frequency of clinic visits.

“I felt just fine… I feel just fine because it gives me strength…No, am okay with the way I always come.” (ADM)

Two ADM arm participants recommended changing the frequency of the clinic visits. One participant reflected on her busy schedule, while another reported a preference for clinic visits once a month instead of weekly.

“I was feeling ok (baby noise) …Yes [would make changes] …(laughing) Busy!! (baby noise) … Because of these busy schedules.” (ADM)

“I feel just fine… No, I would want to change…the way I used to come here like once in a week? I can come according to what you tell me (she laughs) … ah no [weekly], maybe monthly better…yes.” (ADM)

Most (9/11; 82%) of IPT arm participants reported positive attitudes towards clinic visits, stating that they either “felt happy” or “felt good/just fine” about coming to the clinic for counseling sessions. Most participants expressed satisfaction with the frequency of counseling sessions.

“I felt good, there is no problem…It was just fine, because time we were given, the time we spent here doing everything and learning, when it comes to learning what we were supposed to learn, we learnt.” (IPT)

“I felt just fine…they [number of counseling sessions] were just fine…they were just fine but again the cravings are still there.” (IPT)

#### Attitude Toward Counseling Sessions

Nearly all IPT arm participants responded to questions about counseling type (i.e., individual or group counseling) and mode (in-person or by telephone), and frequency of the counseling sessions during the trial.

##### Group Counseling or Individual Therapy

Most (8/9, 89%) IPT arm participants who talked about types of counseling sessions reported group counseling as their preferred type. Two of these participants perceived group counseling as useful for fostering strong communication. Some participants compared counseling type and mode, contrasting telephonic interviews with in-person group counseling.

“The group counseling is better because we are able to sit down and discuss properly and give good advice.” (IPT)

“…both are okay, but better in groups than to talk on phone. Sometimes it doesn’t turn out to be good. Unless when you are seated together as a group, you feel free than on phone, you find that you are on phone and you receive visitors at home. You’ll talk a bit and find that there are people who have come, and children want something, you can’t say am busy. Especially when am with the counselor, I can’t say that am busy. So, it’s better to come here, that’s when you can talk properly. It’s important.” (IPT)

One IPT arm participant who talked about types of counseling sessions reported that group counseling “may be safe if you’re colleagues or know who you’re dealing with,” but some people might be stigmatized if participating in group counseling.

##### Frequency of Counseling Sessions

All (9/9; 100%) IPT arm participants who talked about the frequency of the counseling sessions expressed acceptance of the sessions.

“I think they [frequency of study counseling sessions] are just fine because the excessive thoughts I had are reducing as time goes by.” (IPT)

“In my opinion, I would say that coming frequently is much better because the way we have been assisted is the same way others will be assisted.” (IPT)

##### Attitude Toward Medication Dispensation and Dosage Modifications

ADM participants were asked whether they had to increase their medications over the course of the study, why they had to increase their medication, and their thoughts on whether their medication could have been increased without them coming to the clinic. All 12 ADM participants responded to this question. Three-quarters (9/12; 75%) of these participants reported that their medication increased during the study.

Nearly half (4/9; 44%) of participants with a medication increase reported that they “don’t know” why their medication increased, one reported that her medication increased “because of […] excessive thoughts,” and another one reported that she was told that she had “developed”. Most (7/9; 78%) participants who cited medication increase did not believe medications should be increased without a clinic visit.

“Yes it [medication] increased… because of the excessive thoughts I had (she laughs) … no, you need to come to the clinic [to have medication increased].” (ADM)

### Barriers and Facilitators to Intervention Adherence

#### Medication Adherence

When asked about PPD medication adherence in addition to taking ARVs, all ADM arm participants expressed ease in remembering to take their PPD medication because of their ARVs. While some reported taking their antidepressant with the ARVs, some reported taking them at different times of day. Facilitators to ADM adherence among participants included established ARV medication routines, daily habits, and strategies to remind them to take their medication, including reminders from their HIV clinic. These facilitators promoted medication adherence.

“Uh it was easy because I used to take [ARVs]. So even taking these, it was very easy. So, I used to take them often…Yeah, I would remember [to take Sertraline]. Yeah, I used to remember it because when I take ARVs together with them, so I thought just to help thupi (body). Help my body. I used to take them and say ok when I take these at least then it will help me.” (ADM)

Although none of the participants reported barriers to taking their ADM, one participant shared some difficulties remembering to take her medication initially, until she got used to it.

“It so happens that you get used cause you remember that time that you need to take your [ARV] medication. So you just get used…It’s hard [to remember] at first because you haven’t gotten used. But when you get used, your heart will tell you that you need to take it, so you take, and drink then forget about it.” (ADM)

#### Medication Side Effects

ADM arm participants were asked if they experienced side effects from taking their antidepressant medication and actions taken. Slightly less than half (5/12; 42%) of ADM arm participants reported initial mild medication side effects such as nausea, vomiting, headache, or difficulty sleeping which had since resolved.

“No, there was nothing…just a headache. For two days only…no, just at the beginning. It stopped now.” (ADM)

“It used to give me problems when I first started. But from the time I got used, it stopped. I am now fine, the way I am.” (ADM)

#### Health beliefs and attitudes towards depression and treatment for depression

When asked for their advice to other women with depression, participants expressed a wide range of health beliefs including that encouragement and social support reduce depression, and that psychiatric medications and counseling are valid treatment options for depression. Participants expressed positive attitudes toward the complexities and challenges of depression. Most participants reported providing social support and encouragement to other people with depression. It was also clear from the interviews that participants viewed depression as linked to living with HIV.

“I would encourage my friend to say, “let’s go, be strong,” because these things have happened (HIV status), there is nothing that you can do. You just have to follow the rules that you have been given at the clinic.” (ADM)

“I can encourage them to say that they shouldn’t think too much and know that things happen. Because if you think too much, you find that you die due to excessive thoughts. So, I can encourage them because even me I learnt how to control myself.” (ADM)

“I would give them good advice. Because there is a huge change from the way I was in the beginning. Things have changed, I used to think about a lot of things, even about committing suicide so even now I would give them good advice about how one would live and take care of themselves and so on.” (IPT)

The advice I would give them according to what I was taught at the clinic, I would advise them to say if a person is thinking too much, you need to talk to them properly. We are a lot of people, if one is taking medication and they stop, they take medication and they stop. It happens that you get the medication and drink, and your years move forward. So, don’t follow what others say behind your back, excessive thoughts kill. So just put everything behind, even if they say bad things to you, just forge on and, take your medication and follow the instructions you are given.” (ADM)

One participant expressed a positive attitude toward counseling sessions. She discussed the simplicity of talking to strangers/unfamiliar people. She also described the importance of empathy when counseling people with depression.

“What I would tell them is a lot. There’s a lot to be told. But like you guys helped me just with a few stories, I can’t even remember how many there were. You know every story that you read to me really talked about a part of me. It just takes a stranger like I was telling you. It’s easy to relate. A prophet is not respected in his homeland. It’s the same thing. If I go to a person I know, who is my neighbor, they will say (client name): she doesn’t have a husband, she doesn’t have a job, she doesn’t have a business what can she offer me. But if you showed up, she doesn’t know you or where you’re coming from. But if you talk about…you know you were like cleaning my wounds like you were familiar with my wounds that’s the way this counseling was. So, I could help other women, strangers, not people I’m familiar with, the way you people helped me. By God’s grace even more because of my experience in life. So, it’s easy for me to open up, to associate, to help somebody who doesn’t know me. That person can be told the same thing by her mother that I will go and tell her, but it will make a different impact because I’m a stranger.” (IPT)

#### Combination of Social Support and Medication

A participant in the ADM arm expressed that despite taking medication, some people, including herself, think they’re at risk of dying. With a combination of social support and medication, one can learn to manage and cope with depression. This is suggestive of combined medication and IPT treatment approach among patients with depression.

“I can tell them about the study and what they offer us. We teach them, help get rid of excessive thoughts and encourage them very well or even on medication, if they are taking medication, that’s when we think a lot like I take this medication every day, I’ll still die, you see? But if there is someone beside you, you learn fast.” (ADM)

#### Stigma surrounding HIV and mental health

Nearly all participants responded to the question about the presence of stigma present in their communities. Although participants were asked about stigma around mental illness, participants often conflated mental health and HIV-related stigma and seemed more comfortable addressing questions about stigma around HIV.

##### Social Stigma

Participants reported the existence of stigma in their communities and specifically talked about social stigma, including pervasive stereotypes, discrimination, and public disapproval of mental health conditions. These participants reported that stigma often incited fear, embarrassment, anxiety, and suicidal ideation among affected people in the community.

There are two people in our neighborhood who are [HIV] positive so it’s a situation where you don’t know who you tell someone not knowing they are not trustworthy then that person goes to tell someone else, and she gets to hear about it even if it’s something small. So, it happens that because you have excessive thoughts, all you’ll think of is killing yourself and for sure you can kill yourself. (IPT)

“Mmm…the stigma is there. Especially the embarrassment when they discover your status. The stigma is still there. When they’ve just discovered, just know you’re not going to be free. Unless they don’t know about your status.” (ADM)

“We do hear; 3 quarters of those issues happen. You find that you are having excessive thoughts and there is no one to assist you to get over them. Instead, you find that person making things worse for you. So, in thinking too much, instead of you feeling better, things get worse because of the bad that that person is doing. (ADM)

##### Self-stigma

In addition to social stigma, an IPT arm participant reported struggling with internalized negative stereotypes, leading to shame and hopelessness, that prevented her from discussing her mental health issues with her family members.

Like uhh…it’s very severe. Like a person…can you imagine I was unable to talk to my own siblings. Because I’m worried of what they will say, what they will think, there’s a lot of stigma in the compounds. A lot of it. You know it takes a stick of matches to destroy the whole forest. That’s how it takes an ignorant person to destroy somebody’s concept of things.

#### Approaches to Stigma Reduction for Mental Health

Individual counseling, clinic support, and education and sensitization of the community were cited by participants as approaches to mental health stigma reduction.

##### Counseling

IPT arm participants who reported mental health stigma in their communities, particularly public/social stigma, reported that counseling could help decrease stigma associated with mental health.

“Yes, because we need to decrease stigma because it has killed a lot of people. So we need to start protecting those people by counseling, teaching them how they should live and how to avoid what someone may say. Even though they may talk a lot, we need to be there for that person so that they can be well.” (IPT)

##### Clinic Support to Decrease Stigma

ADM arm participants who had reported mental health stigma in their community and another ADM arm participant who had “…forgotten” whether stigma existed in the community reported that clinic support can help decrease mental health stigma in the community.

“The only people that can manage to encourage them are you people from here at the clinic. Would people in the community encourage us as much as you people at the clinic do? And the way you people here are, you do things privately. You differ from the people in the community. You find that someone will tell you something then you go and start telling the whole world about it. So here at the clinic you managed to encourage and strengthen us looking at the way we were and issues we faced. You treated us like young children listening to us, until that time came that even us we gained strength and those excessive thoughts we had started to go far away.” (ADM)

##### Education and Sensitization of the Community

Three IPT arm participants and two ADM participants reported that education and sensitization of the community would help reduce mental health stigma in the community.

“I think to teach them… Mhm so at least they should understand what mental health is and what causes that. Maybe they can understand.” (IPT)

“…Bring them here and teach them…. that’s when they can understand.” (ADM)

#### Perceptions and disclosure of participating in a research study for treatment of depression

Participants from both arms were further asked to report experiences they have heard from other women participating in the study. Slightly more than three-quarters of participants responded to this question (10/12 (83%) ADM arm and 9/11 (82%) IPT arm.) Over half (6/10; 60%) of ADM arm participants and one IPT arm participant reported not hearing any conversations about the study among participants. Of those who reported hearing perceptions of the study, most in the ADM arm were positive (3/4; 75%) compared to slightly less than half in the IPT arm (4/8; 50%). One ADM and IPT arm participant reported hearing negative perceptions, and two IPT arm participants reported hearing mixed perceptions from others. Another IPT arm participant reported hearing discussions that the study is related to mental health.

##### Positive Perceptions Towards the Study

Participants reported hearing positive and encouraging conversations from other participants. Positive perceptions were related to improved suicidal thoughts, behaviors, mood, and other depression symptoms.

“I heard, they just said that this study is very good. Others said, “I wanted to kill myself, but I stopped”. They teach well here so a lot of them appreciate especially the ones I chat with here.” (ADM)

##### Negative Perceptions Towards the Study

One ADM arm and IPT arm participant reported hearing negative perceptions about the study. The ADM arm participant reported hearing other women discouraging others from taking the study medication, saying it was the same as the medication administered to mentally disturbed women at Chainama Hospital, a public psychiatric hospital in Lusaka. The IPT arm participant reported overhearing some women saying that the study medication makes them hungry and sleepy.

“There those who were saying, ‘you should stop taking the medication, you should throw it. The medication is not good’… They say, ‘the medication is given to the mentally disturbed women at Chainama hospital so you should stop taking it’.” (ADM)

##### Disclosure of Study Participation to Family, Friends, and Partners

Slightly less than two-thirds (7/11; 64%) of ADM arm and slightly over a third (4/11; 36%) of IPT arm participants reported disclosing their study participation to members of their families, including their partners, as encouraged by study staff. Participants from both arms discussed open communication between them and their partners, the educational benefits of the program, and informing their families of their whereabouts as the motivators for their disclosure. Most of these participants reported receiving nothing but support and encouragement from their family members, including their partners.

“Family yes, I told my sister…Yes [told partner] …I just decided that I should tell them, and I told them that there is a program I want to join so they also encouraged me saying it’s okay. It encourages you, that’s the way it’s supposed to be…They felt just fine.” (ADM)

“Yes I did tell them…Yes, I told him [partner], even my parents, including my sisters…From the time I started taking the medication when I became positive, I never wanted to associate with anyone and I used to feel lonely, I just used to feel that loneliness and since I am positive, they will know that I have gone to Kamwala clinic. My family and everyone else need to know that that’s where I have gone to. When am leaving home, they need to know my whereabouts, that where has she gone, she’s gone there. Then he also encourages me about going to the clinic. You just have to go because you learn a lot. Even teaching, I also teach him… he felt good… they were also happy. They were also envious.” (IPT)

One IPT arm participant attributed her decision to discuss participation in the study to stigma. She discussed challenges with cultural and public stigma regarding her family and peers. She reported that the program empowered her to find joy and contentment within herself. Furthermore, she used lessons learned to welcome dialogue about the program.

“Yes, I did, I told my sister…I think I told everybody…The first time I thought it was weird. Why should I tell them? You know some family members never knew I was positive. […] You know if something happens here at the clinic you tell somebody in the compound then it will be all sorts of stories. So before I go to that I want to hear [my sister’s] point of view. […] So she took me here and I heard about this and I told her, she said I think it’s a very good program and it will be helpful because she’s the person closest to me. She saw what I was going through. […] After she agreed I started opening up, going out in groups, like to parties. People would notice, like a cousin would come up to me and say ah what’s happening to you. […] In our family, the only time you get respected is either when you’re working, you’re educated and you’re working. […] You’re not begging from anyone. So that’s the only time they could respect you but mostly they count on somebody who is in a relationship hoping they’ll say they’ll get married and settle down. I would say do you know that I just learned that I don’t need a man in my life to be happy? I don’t need anybody’s permission to be happy, except the creators. As long as he has given me life he has given me permission to be happy and grateful. So that’s how I went on telling them about this program. When somebody’s asking about why the sudden change. That’s how I taught them about it.” (IPT)

“I told my family members and my friends. Everyone knows that am in this program. I also encourage them saying when you are taking ARVs, you are not supposed to be thinking about these things…Yes, I did [told partner], I also told others. Even when am leaving home I mention to him that am going to the study clinic for counseling sessions…I decided to tell him because I saw something good in this program. That’s why I informed him for him to be aware of the program so that he also sees something good in this in it…He felt very good about it [study participation].” (IPT)

##### Non-disclosure of Study Participation to Partner, Family, or Friends

One ADM and two IPT arm participants reported not disclosing their study participation with their partners. One of the IPT arm participants reported that her partner once disapproved of her involvement in another study, and therefore decided not to disclose her participation in this study.

“No [did not tell partner] …there was a time that I joined a program at Chawama clinic. It brought a lot of noise because I used to receive transport money, he thought as if I was just going there for money… yes (she laughs) so during this time I did not tell him.” (IPT)

One ADM arm participant reported non-disclosure of her study participation to anyone, for fear of getting stigmatized, materializing from anticipated gossiping.

“No, I did not tell anyone… he also doesn’t know… I just decided that it should remain my secret…yes, because when you tell someone something and you get into a disagreement, they start revealing things and they start gossiping about you just like that.” (ADM)

## Discussion

Our study, which randomized postpartum women living with HIV and depression to either interpersonal counseling or antidepressant medication, found that most women reported acceptability of the treatment arm to which they were assigned and high perceived efficacy of the intervention. Women expressed that either IPT or ADM was appropriate for treating depression and emphasized the role of social support and encouragement that they received at the clinics. Women also reported learning about depression, managing intrusive thoughts, self-care, and reaching out for help. Finally, many women expressed feeling stigmatized, often conflating stigma related to HIV and stigma related to mental health.

Consistent with our findings, several studies in SSA have found high acceptability for psychosocial treatment of depression, including interpersonal counseling, motivational interviewing, and problem-solving therapy [22–24]. Some women in our study reported that group counseling might foster strong communication while others expressed concerns surrounding privacy, especially if the counselor were a member of her community. This is consistent with a qualitative study among women with perinatal depression in Malawi in which participants preferred individual to group therapy [24, 25]. Future studies should nonetheless evaluate the acceptability of group IPT in Lusaka, as studies in Tanzania and Kenya have shown group therapy to be both feasible and acceptable among women with HIV and depression [26, 27]. Since our study occurred during the pandemic, telephonic counseling was attempted during weeks when participants could not come to the clinic. Despite a shift towards technology-based services for mental health care throughout the continent, we found that therapy via telephone was not acceptable in our population due to lack of privacy and competing responsibilities at home.

Few studies in LMICs report use of antidepressant medications to treat postpartum women with depression [28]. Consistent with findings from a medication-based treatment study in Malawi, our study found that antidepressant medication was highly acceptable to women randomized to that arm [29]. All women randomized to the ADM arm had their medication increased over the course of the study, following standard clinical algorithms that seek to balance side effects and treatment efficacy. Notably, half reported that they did not know why their dosage changed, indicating that more efforts are needed to train providers to effectively communicate medication-based treatments changes to their patients. All ADM arm participants reported high medication adherence citing their established ARV medication routine as a motivator and reminder for their adherence. Studies on the acceptability of ADM treatment for depression among adults with HIV in SSA differ in their findings. Some report medication-based treatment is acceptable to patients, while others report challenges with medication-based therapy in low-resource settings, including limited knowledge of medication-based treatments for depression among patients and providers, challenges with reliably accessing medications, and a preference for psychosocial interventions [25, 30–32]. This study provided participants with medications, and we suspect the same barriers to access are present in Zambia. Furthermore, though participants reported high acceptability of ADM treatment, one participant described stigma associated with antidepressant medication treatment in her community. As stigma can affect medication adherence, community education on depression and its treatments may further improve treatment outcomes.

Another theme that emerged through our interviews was that despite widespread use of antiretrovirals and HIV becoming a chronic disease, HIV-related stigma remains highly prevalent in Zambia, contributing to anxiety and depression among many of the participants in our study. We attempted to disentangle stigma from HIV versus stigma related to anxiety and depression and most women conflated HIV and depression related stigma in their responses. Consistent with literature from SSA, women identified social support and psychotherapy as factors that improved coping skills around dealing with stigma [33].

### Study Limitations

Less than half of the participants in our parent trial were included in this study; thus, our study participants may not have been representative of the trial population. Although baseline characteristics were similar on the measures in Table 1, they may have differed in unmeasured characteristics as women self-selected to participate in the semi-structured interviews. We additionally noticed that certain questions were difficult for participants to understand, mostly due to question framing. Specifically, attitudes towards counseling session mode and type were asked as a single compound question (i.e., “What would you think about having the counseling sessions on the phone or in groups?”), as if participants were being asked to choose between mode and type of counseling sessions. During analysis and results interpretation, we separated the responses into mode and type of counseling sessions. This limited our ability to assess the acceptability of group therapy among this population. Though interviewers were native speakers in the language of participants’ preferred language, we noted some participants had difficulty understanding translated interview guides, potentially due to differences in proficiency with formal dialects between study staff and participants. Finally, interviews were conducted by study team members, which may have introduced a desirability bias, as participants may not have wanted to express reservations regarding study procedures.

## Conclusion

Zambian women living with HIV participating in a pilot feasibility study of treatment for postpartum depression had generally positive attitudes towards the treatment of depression and reported high perceived efficacy of their assigned interventions. Participants from both arms had positive perceptions of participating in the study and found both treatment options acceptable. Further studies are needed to explore the acceptability of group vs. individual therapy and telephonic counseling in this setting. Nonetheless, these findings support the conclusion that a full-scale randomized trial to evaluate the effect of treating postpartum depression on maternal and child HIV outcomes is both feasible and acceptable among women in Lusaka.

### Supplementary Information

Below is the link to the electronic supplementary material.

Supplementary material 1 (DOCX 26.5 KB) Online Resource 1a: Follow up SSI Interview Guide_IPT arm—v1.0 24 oct 2019

Supplementary material 2 (DOCX 26.7 KB) Online Resource 1b: Follow up SSI Interview Guide_ADM arm—v1.0 24 Oct 2019

## Data Availability

Deidentified data are available from authors upon request.

## Code Availability

The qualitative structural code book is available from authors upon request.
