# Supplementary material for: Acceptability of a Randomized Trial of Anti-depressant Medication or Interpersonal Therapy for Treatment of Perinatal Depression in Women with HIV
Source: AIDS Behav. 2024 Feb 14;28(4):1123–36. doi: 10.1007/s10461-023-04264-0 (PMC10940463; doi:10.1007/s10461-023-04264-0)
Supplement: Supplementary file 2 — Supplementary material 2 (DOCX 26.7 KB) Online Resource 1b: Follow up SSI Interview Guide_ADM arm—v1.0 24 Oct 2019 [file 10461_2023_4264_MOESM2_ESM.docx]

# Supplementary Material 1b: Interview Guide for Participants in PDS Trial – ADM Arm

**Manuscript Title**: Acceptability of a randomized trial of anti-depressant medication or interpersonal therapy for treatment of perinatal depression in women with HIV

**Journal**: AIDS and Behavior

**Authors**: M. Bridget Spelke^1, 2§^, Eunice Okumu^3^, Nzi R. Perry^3^, Bryan S. Blette^4^, Ravi Paul^5^, Crystal E. Schiller^6^, J.M. Ncheka^5^, Margaret P. Kasaro^2^, Joan T. Price^1,2^, Samantha Meltzer-Brody^6^, Jeffrey S.A. Stringer^1, 2^, Elizabeth M. Stringer^1, 2^

^1^ Department of Obstetrics and Gynecology, University of North Carolina School of Medicine, Chapel Hill, United States

^2^ University of North Carolina – Global Projects Zambia, Lusaka, Zambia

^3^ Social and Behavioral Science Core, Center for AIDS Research, University of North Carolina, Chapel Hill, United States

^4^Department of Biostatistics, Vanderbilt University Medical Center, Nashville, United States

^5^ Department of Psychiatry, University of Zambia School of Medicine, Lusaka, Zambia

^6^ Department of Psychiatry, University of North Carolina School of Medicine, Chapel Hill, United States

^§^ Corresponding author: M. Bridget Spelke

348 Independence Ave

Lusaka, 10101, Zambia

Phone: +260 763885942

Email: [bspelke@email.unc.edu](mailto:bspelke@email.unc.edu)

# Interview Guide for Interviews with Women Enrolled in Trial – ADM

Introduction

Hello, my name is and I’m working with University of North Carolina – Global Projects Zambia (UNC-GPZ) organization. Thank you for agreeing to talk to me today. As part of this study, we are interested in hearing your experiences with taking part in this research study about treating postpartum depression. We are hoping that by understanding what women go through when they take part in research, we can improve the way we do studies in the future.

I would like to audio record the interview, so I don’t miss anything that you say and so that later I can be sure that I don’t misunderstand what you said. The recording of this interview will be written down and then the tapes will be destroyed. Your name will not be included in the audio recording or on any of the documents so that no one will know that you took part in this interview. Your answers will be kept confidential and secure. Is it okay if I audio record our discussion today?

Thank you.

Your participation is completely voluntary. If you want to stop at any time or do not want to answer a question that I ask, just tell me. That is not a problem. I am very interested in your thoughts and opinions. There are no correct or incorrect answers. Please feel free to say whatever you think. The interview will last about 30 minutes. Do you have any questions before we start?

Questions in italics are required.

# Introductory question: 2 min

Good morning. How are you? How is your family?

# General questions on study and medication-20 min

1. *Now that you have been enrolled in the study for some time, can you tell me how you are feeling since you were enrolled in the study?*

Probe: Do you think the medication worked? Have other things happened that may have affected your mood?

Probe: Please describe any times your thoughts on taking the medication have changed while participating in this study.

1. *How did you feel about coming to the clinic to pick up your medicine? What changes would you make in the number of visits to the clinic and why?*
2. *In your opinion, how has the medication made you feel? Describe how easy or difficult it has been to perform your daily activities and responsibilities such as cleaning or cooking since you started taking the medicine. In what ways has your ability to perform your normal behaviors changed or remained the same?*
3. *Can you explain if you have had any side effects from taking the medicine. If you did have side effects what did you do to make them go away? Did you have to stop the medicine or lower the dose?*
4. Over the course of the study, have you had to increase your medications? Why did you have to increase your medicine? Do you think your medicines could have been increased without coming to the clinic?
5. *What was it like having to remember to take these medications, when you are already taking other medications for your HIV?*

Probe: How did already taking other medications make it easier or harder to remember these new medications?

1. *How did you feel when you learned that you had been not been chosen to receive counseling through the study? Please explain.*

# Experience in study-25min

- 1. *Did you tell any of your friends or family members that you are taking part in this study? Did you tell your partner about the study?*

Probe: If you decided to tell them, how did you decide whether to tell them?

Probe: What did they say when you told them about being a part of this study? Probe: How did they feel about you being part of the study?

Probe: If you decided not to tell them, why did you not want to tell them?

- 1. *What kind of experiences have you heard from other women participating in the study?*
  2. *What kind of stigma do people in your community experience with regard to mental health issues? Can you describe things that can be done in the community to decrease stigma associated with mental health*? *Probe: What sorts of things?*
  3. Did you learn anything from being in this study? If so, what did you learn?
  4. *After being in the study, what would you tell other women who may have depression?*
